# Understanding the feasibility and environmental effectiveness of a pilot postal inhaler recovery and recycling scheme

**DOI:** 10.1038/s41533-023-00327-w

**Published:** 2023-01-21

**Authors:** Anna Murphy, David Howlett, Aaron Gowson, Harriet Lewis

**Affiliations:** 1 University Hospitals of Leicester NHS Trust, Leicester, UK; 2 PharmaDelivery Solutions Ltd, Norfolk, UK; 3 Chiesi Limited, Manchester, UK

**Keywords:** Health policy, Asthma, Chronic obstructive pulmonary disease, Patient education

## Abstract

All inhalers have an environmental impact; the majority are not recycled, with many disposed of inappropriately through domestic waste. To assess the feasibility of a method for recovering and recycling inhalers, Chiesi Limited (Chiesi) set up and funded ‘Take AIR (Action for Inhaler Recycling)’, a 12-month pilot postal scheme facilitated by community pharmacies across Leicester, Leicestershire, and Rutland, and hospitals in Leicestershire. All inhalers were accepted in the scheme. The recovered pressurised metered-dose inhalers (pMDIs) were dismantled and component parts recycled where possible; the remaining propellant gas was extracted for reuse in refrigeration and air conditioning industries. Other inhaler types were incinerated in an ‘energy-from-waste’ facility. From February 2021 to February 2022, 20,049 inhalers were returned; most (77%) were pMDIs. So far, Take AIR has saved the equivalent of an estimated 119.3 tonnes of carbon dioxide emissions from entering the atmosphere. Our experience demonstrates the feasibility and effectiveness of a postal inhaler recovery and recycling scheme, which could be used as a foundation to build future initiatives.

## Introduction

A broad range of inhaler devices and drug combinations is available for the management of respiratory conditions such as asthma and chronic obstructive pulmonary disease (COPD)^1^. Inhaler device types most commonly used for asthma and COPD are pressurised metered-dose inhalers (pMDIs) and dry-powder inhalers (DPIs)^1,2^. Soft-mist inhalers (SMIs) are also used but predominantly for the treatment of COPD^2^. It is important to have a range of inhaler devices and drug combinations available to patients because all options have advantages and limitations^1^. A range of factors such as age, comorbidities, inspiratory rate, and importantly, inhaler technique can affect how well an inhaler device suits a patient^1,3–6^. Matching each patient to the correct medication for effective disease management is a challenging process, and different aspects such as patient engagement, medication, and device should be considered in this decision^7^. One important aspect is patient satisfaction with their device, which can significantly improve adherence and could result in improved disease management^8,9^.

The prevalence of inhaler prescriptions by type varies significantly by country^10^. For example, in 2017, 70% of all inhalers sold in England were pMDIs, compared with just 13% in Sweden^11^. Countries around the world also have different practices of medication disposal^12^. Regardless of inhaler type, the use and disposal of all inhalers has an environmental impact, with the primary contribution in pMDIs arising from the fluorinated propellant gas (F-gas), a known greenhouse gas (GHG)^13^. The pre-disposal impact of manufacturing the materials used in inhaler production, such as steel and aluminium, is also a consideration. pMDIs use hydrofluorocarbons (HFCs) as the propellant, predominantly HFC 134a and HFC 227ea^14–17^. HFCs are part of the broader group of F-gases that, together, are responsible for ~2% of total, global GHG emissions^16,18^. Although most GHG emissions from HFCs come from the air conditioning and refrigeration industries (rather than pharmaceutical products), HFC 134a and HFC 227ea have a global warming potential substantially higher than that of carbon dioxide (~1000 and ~3000 times higher, respectively)^16,18,19^. In addition, all pMDIs are manufactured with an overfill of medication (including propellant) to ensure reliable delivery of doses, such that even inhalers with all labelled doses used will have a pre-determined amount of propellant still remaining in the inhaler at disposal (as determined by the manufacturer)^20^. Inhalers are responsible for 3% of carbon emissions from the UK National Health Service (NHS)^21^.

Although inhalers consist of potentially recyclable materials (including plastics, metals, and the propellants), they are often disposed of in domestic waste. Some may be returned to community pharmacists, where they are disposed of as clinical waste via the service for all unwanted medicines (provided through the NHS contractual framework)^22^. Qualitative data from a survey of 100 patients with COPD in Leicester revealed that 93% of patients dispose of their inhaler in household waste^23^. Similar findings were evident in a survey of 100 patients at Hillview Surgery in London, which found that 98% of patients put their inhaler in domestic waste^24^.

At present, there are limited systems in place to collect and recover used and/or unwanted inhalers. Furthermore, some patients may not be aware that the pharmacy take-back service for unwanted medicines^22^ is available, may not consider their inhalers as medicines, and/or may assume their inhalers are empty, and can, therefore, be discarded in the domestic waste.

The NHS has set targets to reduce carbon emissions as stated in *Delivering a Net Zero NHS*^21,25^. The NHS Net Zero target includes reducing its carbon footprint by 80% from 1990 levels by 2028–2032^21^. The plan includes a goal to reduce carbon emissions from propellant in pMDIs by increasing the use of DPIs (which do not contain propellant), increasing the greener disposal of used inhalers, and supporting innovation in and use of lower carbon propellants and alternatives^21^. A 2018 report from the UK Environmental Audit Committee on reducing F-gas emissions states that ‘medical companies or the NHS should establish a pharmacy-based inhaler recycling system to ensure that residual HFCs from pMDIs are recycled rather than released in landfill.’

To contribute towards the holistic goal of reducing the environmental impact of inhalers, with a focus on reducing the propellant-associated carbon impact of pMDIs, Chiesi set up and funded a pilot postal inhaler recovery and recycling scheme.

**Fig. 1:**
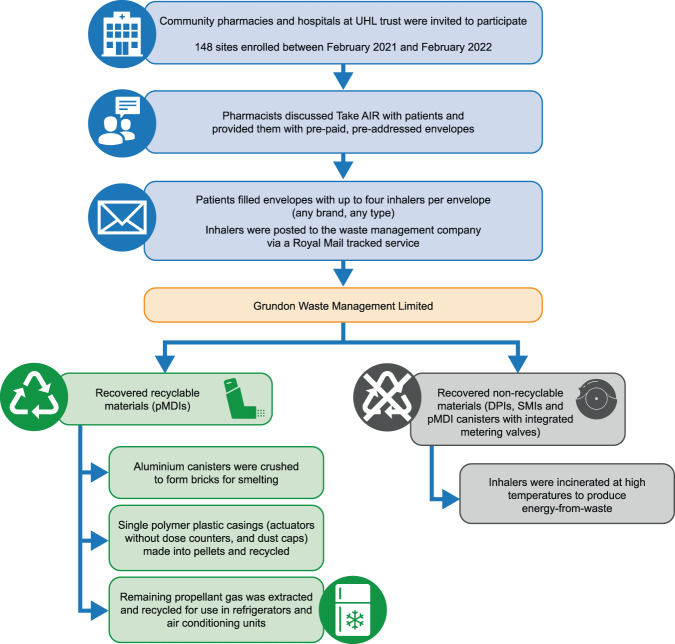
The Take AIR Scheme: study design and recycling process. AIR action for inhaler recycling; DPIs dry-powder inhalers; pMDIs pressurised metered-dose inhalers; SMIs soft-mist inhalers; UHL University Hospitals of Leicester (Leicester General Hospital, Glenfield Hospital, Leicester Royal Infirmary).

Previous inhaler recycling schemes have relied on inhalers being dropped off at community pharmacy collection points. GlaxoSmithKline funded a recycling scheme (GSK Complete the Cycle) based on patients returning their inhalers to their community pharmacy^26^ and Teva Pharmaceuticals funded a similar scheme^27^; however, there is a lack of publicly available information about the current status of these schemes. To test the feasibility of an alternative solution for recovery and recycling of inhalers, Chiesi set up ‘Take AIR (Action for Inhaler Recycling)’, a 12-month pilot postal scheme across Leicester, Leicestershire, and Rutland (LLR). The Take AIR scheme was designed and funded by Chiesi with a primary aim of developing and assessing the feasibility of a postal option for patients to return inhalers for recycling, as an alternative to the community pharmacy waste collection service (Fig. 1). Other key aims of the scheme were to divert used or unwanted inhalers from domestic waste streams, to reduce the emissions into the atmosphere of GHGs contained in the pMDI canisters, and to estimate the carbon footprint per inhaler and the resulting effects of the scheme in terms of reduced carbon emissions using predictive modelling. Here we present the results from the Take AIR scheme.

## Results

### Engagement from pharmacies and patients

**Fig. 2:**
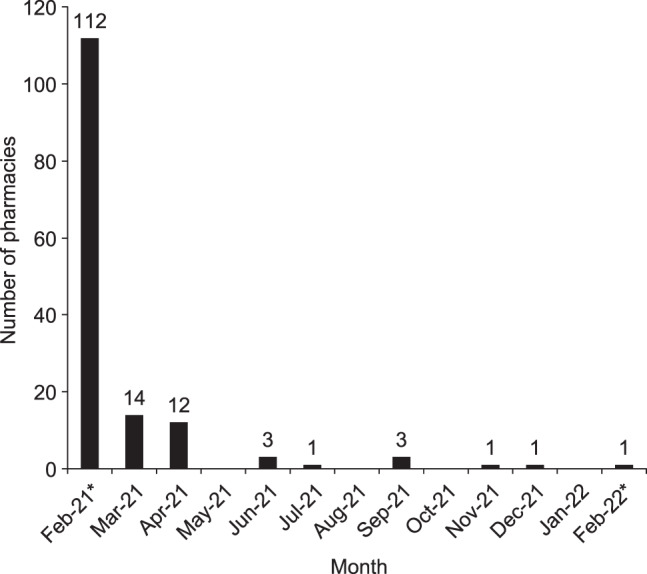
Number of pharmacies enrolled in Take AIR in each month. *February 2021 and February 2022 data include only 2 weeks of the month.

**Fig. 3:**
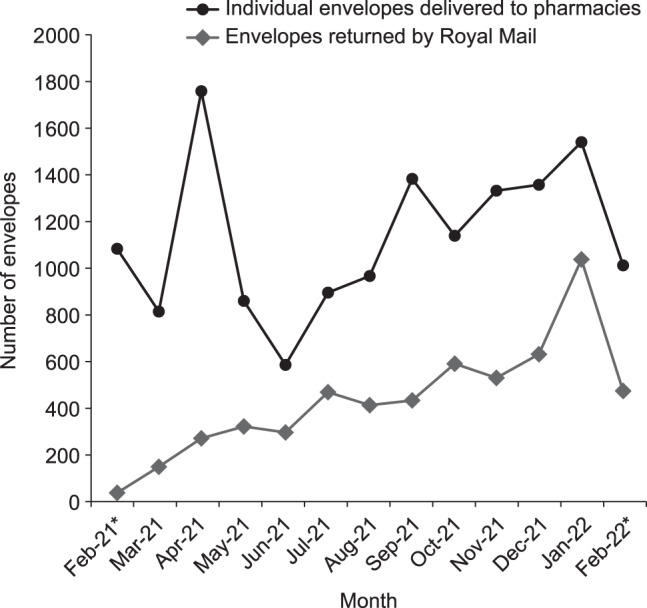
Envelopes delivered to pharmacies and envelopes returned by Royal Mail in each month. *February 2021 and February 2022 data include only 2 weeks of the month.

In the first 12 months, 148/227 pharmacies (65%) and the UHL NHS Trust (three hospitals, coordinated by one site) voluntarily enrolled in the Take AIR scheme (Fig. 1); 76% of the sites enrolled in the first 2 weeks (Fig. 2). In total, 14,805 envelopes were delivered to pharmacies, of which 5686 envelopes were returned to the waste management company via Royal Mail. Although there were fluctuations in pharmacies ordering and receiving envelopes month-to-month, there was a steady increase over the study period in the number of envelopes being returned from patients via Royal Mail (Fig. 3). The number of envelopes returned increased seven-fold from 149 envelopes in the first complete month (March 2021) to 1041 envelopes in the last complete month (January 2022).

### Inhalers returned to waste management

**Fig. 4:**
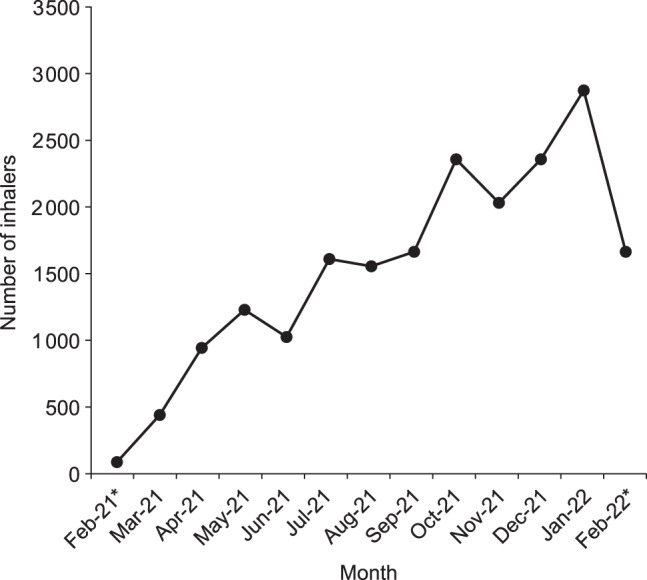
Inhalers returned to waste management in each month. *February 2021 and February 2022 data include only 2 weeks of the month.

By the end of the 12-month period, 20,049 inhalers had been returned to the waste management centre in envelopes posted by patients; on average there were 3.5 inhalers per envelope. This represents an average weekly return rate of 386 inhalers, equivalent to an estimated 2% of all inhalers prescribed in the LLR region (based on an estimated 20,179.6 prescribed inhalers in total per week). As with the number of envelopes returned by Royal Mail, the number of inhalers returned to the waste management facility increased over time (Fig. 4). There were 451 inhalers returned to the waste management facility in the first full month of Take AIR (March 2021). This increased to 2906 inhalers in the last full month (January 2022), corresponding to an estimated 3.3% of all inhalers prescribed across the LLR region. Most inhalers (77%) returned to the waste management facility were pMDIs and there was little variation month by month in the types of inhalers returned (range for the proportion of pMDIs: 67–82%).

### Carbon dioxide savings

In total, calculations estimated that Take AIR saved a minimum of 119.3 tonnes of carbon emissions from entering the atmosphere. This is equivalent to the carbon dioxide sequestered by 1973 tree seedlings grown for 10 years or 141 acres of US forestry in 1 year. These results should be considered a potential underestimate, as only the manufacturers’ overfill was used for the calculations and any further remaining propellant from the incomplete use of inhalers was not accounted for.

### Response from Take AIR participants

There were 49 responses to the online survey, with 48 patients completing all questions. The survey highlighted that most patients (94%; 45/48) wanted to take part in the recycling scheme because they were concerned about the environment. From the feedback on the scheme itself, the Take AIR scheme appeared to be well received by patients; 90% (44/49) were very satisfied or satisfied with the scheme, and all patients (49/49) thought the instructions around the scheme were easy or very easy to follow. All patients who responded to the relevant question (48/48) thought that the scheme should be available nationally across the UK.

## Discussion

Many inhalers are currently disposed of either in household waste, or through the unwanted medicines collection service at community pharmacies (clinical waste incineration)^22^, with a resulting impact on the environment. These initial 12-month findings from the Chiesi-funded Take AIR pilot scheme demonstrate the feasibility and effectiveness of postal inhaler recovery and recycling as a potential solution for reducing GHG emissions from inhaler disposal.

Take AIR was quickly adopted by community pharmacies in Leicestershire, with the majority enrolling within the first month of the scheme. Uptake by patients was gradual and increased over time, with the increase in inhalers returned (451 in the first full month to 2906 in the last) potentially suggesting more regular use by patients once they became familiar with the scheme and incorporated the returns into their routine. Behaviour change and normalisation of recycling are recognised as key factors for successful recycling campaigns^28^. Indeed, preliminary data from the continuation of Take AIR confirm the increasing trend in returned inhalers, suggesting long-term feasibility and indicating that it would be possible to build on the initial proof of concept via increased collaboration and involvement of multiple stakeholders. Moreover, feedback from participants in Take AIR was positive and suggested that the scheme would continue to be used if made available nationally (though it should be acknowledged that the survey sample population was small).

Take AIR was designed to explore a postal inhaler recycling scheme; the previous ‘Complete the Cycle’ programme by GSK and the ongoing inhaler recycling programme by Teva Pharmaceuticals were both set up as drop-off schemes. Although publicly available data from ‘Complete the Cycle’ are sparse, it was estimated that there were more than 2 million inhalers returned over the 9 years the scheme was open^29^. Teva Pharmaceuticals does not publicly report their return data but does state that 100 pharmacies across Ireland are involved in their scheme^30^. These data from ‘Complete the Cycle’, Teva’s scheme, and Take AIR suggest that there is a willingness to adopt a nationwide recycling scheme if it were to be offered. Specialised recycling schemes for hard-to-recycle products are not novel; other product manufacturers provide drop-off or postal recycling schemes. To help to reduce plastic waste, Novo Nordisk has introduced ‘take-back’ pilot schemes to recycle used insulin pens, with projects in Denmark, the UK, and Brazil, and more countries launching soon^31^. Other non-medical companies with initiatives include the coffee company Nespresso® and printer ink cartridge companies^32–38^. Expertise and learnings from existing and previous recycling schemes can be used to develop and to enhance any future initiatives. For example, in future inhaler recycling initiatives, it could be considered to offer both drop-off and postal options (similar to the Nespresso recycling scheme) to optimise the return rates of inhaler devices.

To enhance the success of any medical recycling scheme, patient education should be a key focus. Patients need to be aware that their inhaler is a medicine and should be treated as such, and not disposed of in domestic household waste or domestic recycling. In this pilot, pharmacies were provided with information about the recycling scheme through the launch communication materials, and information was conveyed to patients upon collecting their inhaler at the pharmacy or at discharge from hospital. In future initiatives, education could be built into the patient pathway earlier (e.g., during consultations with general practitioners and other healthcare professionals, time permitting) to facilitate multiple conversations around inhaler recycling. This approach would provide opportunities for patients to understand fully the importance of correctly disposing of their inhaler. In turn, having a greater awareness of the need for appropriate recycling may give patients a new respect for their inhaler as a medical device and help to incentivise them in the overall management of their condition.

The estimate that a minimum of 119.3 tonnes of carbon dioxide emissions were prevented from entering the atmosphere over the initial 12 months demonstrates the potential for an extension of the scheme to make a notable impact on the reduction of GHG emissions from inhalers. Correct and environmentally friendly disposal of inhalers is not the only way in which the environmental impact of inhalers could be reduced; efficient and effective inhaler use by patients can also reduce the overall contribution to GHG emissions^17,39^. Poor inhaler technique can lower drug deposition to the lungs, leading to higher levels of medication waste^40^, and a lack of awareness around number of doses per inhaler may also lead to patients disposing of their inhaler with surplus medication and propellant remaining. Additional investigation of the weights of inhalers returned will enable real-world assessment of the extent to which patients are disposing of partly used inhalers. Studies have shown that patient satisfaction with their device and drug combination can improve adherence and could result in improved disease management^8,9^. With better adherence, it can be assumed that there would be less inhaler drug waste. To guide the selection of the right inhaler for a patient (one that they can use effectively), the National Institute for Health and Care Excellence has developed a patient decision aid, with information to help patients and healthcare professionals discuss the options for inhalers, taking into account the carbon footprint associated with each type of inhaler^41^. According to the NHS Investment and Impact Fund, one of the suggested interventions to improve respiratory care and health outcomes for patients with asthma and to decrease avoidable carbon emissions includes reducing excessive use of short-acting β2 agonist (SABA) reliever inhalers^42^. To address SABA overuse, the Global Initiative for Asthma Strategy outlines that adolescents and adults should not be treated solely with SABA inhalers and that regular assessments of SABA use (and potential overuse) be conducted on a regular basis^43^.

The NHS Net Zero plan recommends significantly increasing the use of DPIs because these have significantly lower carbon emissions than other inhaler types^44^. Despite the evidence that DPIs have a lower global warming potential than pMDIs, it is also crucial to consider patient needs and preferences when making inhaler decisions^4,11,16,17,45^. A patient’s inspiratory flow rate may determine whether a pMDI is more suited than a DPI; however, other factors such as a patient’s age and existing comorbidities can affect how well they are able to handle a specific inhaler type^3–5^. A future solution to reduce GHG emissions from pMDIs, while maintaining their use in those patients who prefer them, will be to transition to low global warming-potential propellants such as HFA 152a, which are expected by 2025 and are being developed by several companies, including Chiesi^46–48^. However, it is anticipated that not all pMDIs will be transitioned^17^, and inhaler disposal and recycling schemes will be required.

Although the Take AIR scheme could be deemed successful, it should be noted that this scheme was limited to the LLR region and was only run for a defined period of time. In addition, it is worth noting that calculations of carbon emissions are estimates based on the assumption of correct use (pMDI will have emitted all doses as per the label claim); therefore, further research is needed to confirm the validity of the model. A related study is ongoing to investigate the contents of returned inhalers in more detail, with pMDIs that are returned to the waste management company being weighed to evaluate how frequently they are returned unused or partly used. It is also important to consider that there are multiple barriers to overcome when setting up a recycling scheme, including working in line with regulatory, waste, medicine, and postal carriage regulations, as well as identifying the relevant contacts in each agency and department with whom to discuss the scheme. Establishing contact with appropriate agencies for advice was challenging. This advice was required for correct completion of the complex waste transfer notes to move waste via post, as well as agreeing on a suitable low-risk solution for posting inhalers (with potentially flammable materials); four inhalers per envelope was considered low risk. There was also a requirement to align with the Royal Mail’s conditions of carriage. Furthermore, there were challenges with finding suitable and compliant drop-off options for inhalers in commonly used sites, for example, supermarkets; additionally, pharmacies may have limited space to collect inhaler waste and limited resource to sort inhaler waste from general medicines waste. This was the rationale behind Take AIR being set up purely as a postal scheme. Such challenges would need to be considered for any future recycling schemes. Although the Take AIR scheme diverted inhalers from landfill, not all inhaler devices and medicines (DPIs, for example) could be recycled and/or reused; therefore, they still made some contribution to carbon emissions via incineration in the ‘energy-from-waste’ process. Finally, there are no publicly available data on inhalers returned to community pharmacies through existing collection schemes; therefore, a conclusion could not be drawn on whether more inhalers are returned through the postal scheme versus drop-off at community pharmacies.

The coronavirus disease (COVID-19) pandemic presented multiple challenges during this pilot, including the likelihood that community pharmacies may have had reduced capacity to facilitate the scheme and to discuss it with patients. Despite the pandemic, there was still high voluntary uptake from pharmacies, implying a high level of interest in inhaler recycling within the healthcare community. This scheme highlights the important role that pharmacists can play in supporting the education of patients on reducing carbon emissions arising from inhalers that have been disposed of incorrectly.

Scaling up the recycling scheme nationally to make it sustainable in the long term would likely require input from various stakeholders across the country. This would need collaboration between local councils, the NHS, pharmaceutical companies, and waste management partners. Despite the complexity of setting up a nationally available inhaler recycling scheme, the initial findings from Take AIR indicate that it is likely such a scheme would be used by healthcare professionals and patients, and that resulting carbon emission savings could be substantial.

In conclusion, the Take AIR scheme has demonstrated the feasibility and effectiveness of a postal inhaler recovery and recycling scheme for reducing GHG emissions and the environmental impact of inhalers, as well as an encouraging level of participation by pharmacists and patients. Despite the challenges of the COVID-19 pandemic, the scheme provides a proof of principle that can be built on in future initiatives and demonstrates that patients are willing to recycle their inhalers when the process is convenient and easy to use.

## Methods

### Design of the recycling initiative

To inform the design of the Take AIR scheme, a desk-based market research exercise was conducted by Chiesi to evaluate recycling schemes from other industries, patient views, potential achievable carbon savings, and current facilities and processes. Based on the findings from the research, it was essential that the scheme should be easy to use, supported by effective communications from the sponsoring company, and had conveniently located drop-off points (for those products that were not returned via a postal scheme). To assess patient opinions on recycling of inhalers, Chiesi conducted a preliminary UK-based survey of 487 people with asthma who use inhalers. The results indicated that patients would engage with a recycling scheme if it was accessible and easy. Of the respondents, 80% would recycle their used inhalers if they knew more about the available options, 77% would recycle them regularly if they could post them to a recycling centre, and 70% would feel able to contribute towards reducing climate change via the scheme.

Chiesi established a multidisciplinary steering group, with engagement across the LLR health system. The steering group held regular progress and planning meetings and worked closely with a communications agency to provide regular newsletters, mailings, patient feedback, and reports to keep all stakeholders informed of progress.

Community pharmacies were selected to participate in the Take AIR service, and were encouraged to promote the scheme, offer the envelopes to patients at the point of dispensing inhalers, and provide information to support patients. Pharmacies were considered ideal because they provide medicines to patients, either in the pharmacy or via home delivery, and pharmacy teams are in a suitable position to support patients around inhaler use, inhaler technique, and safe disposal or recycling. This is in accordance with one of the quality criteria within the current Pharmacy Quality Scheme, which includes encouraging the return of unwanted and used inhalers for disposal to protect the environment^25,49^.

In February 2021, all community pharmacies in the LLR region (a total of 227) were invited to participate in the Take AIR service by the LLR Local Pharmaceutical Committee (LPC). Pharmacies were encouraged to participate on a voluntary basis and were not financially incentivised to join this scheme or provided with any fee for participating.

Patients were informed about the Take AIR scheme by local pharmacy teams across the LLR area. To facilitate participation, pharmacies were given the flexibility to provide the envelopes to interested individuals at any time that was appropriate, whether proactively (for example, during inhaler dispensing or separate discussions with patients) or reactively upon request from the patient. Each pre-paid, pre-addressed Take AIR envelope included an information leaflet with a QR code directing to a patient feedback questionnaire and a weblink to the Take AIR scheme website. At home, patients were instructed to remove all identifiable information from the inhalers, fill the envelope with up to four devices, seal the envelope, and post it in a letter box. For the pilot period, postage for 25,000 pre-paid envelopes was funded by Chiesi through a tracked postal service with Royal Mail. The number of inhalers per envelope was capped at four devices to stop envelopes becoming too large to fit into letter boxes. Any inhaler of any brand or type could be returned through this scheme.

All envelopes were returned by Royal Mail directly to the contracted waste management company, Grundon Waste Management Limited. At Grundon, the envelopes were opened, and the contents sorted and separated into two waste streams: recyclable and non-recyclable. For pMDIs, the aluminium canisters were crushed to form bricks for smelting. During the crushing process, any remaining propellant gas was extracted to be reused in non-pharmaceutical items such as refrigerators and air conditioning units. The plastic casings (dust caps and actuators without dose counters, made of single polymer plastic) were pelletised to be recycled into other plastic products. All other inhaler types, including DPIs and SMIs, were classed as non-recyclable because of their complexity. These inhalers, as well as pMDI canisters with integrated dose counters (those not easily separated from the canister), were incinerated at high temperature to create energy, using a waste process known as ‘energy-from-waste’.

In October 2021, following the successful launch of the pilot, the Take AIR scheme was extended to three hospitals at the University Hospitals of Leicester (UHL) NHS Trust (Leicester General Hospital, Glenfield Hospital, and Leicester Royal Infirmary), with these sites also providing envelopes. Patients in UHL who were prescribed an inhaler in hospital had the recycling scheme explained to them when discharged, and they were given a pre-paid scheme envelope to use. If patients required more envelopes, these could be collected at any participating pharmacy.

### Analysis of returned inhalers

The waste management partner collected data on what was being returned. The data from the Take AIR scheme presented here were collected over a 12-month period from 15 February 2021 (initiation of the recycling scheme) to 18 February 2022. Using predictive modelling, the amount of propellant in recycled pMDI inhalers was estimated and used to calculate the carbon footprint per inhaler and the resulting impact of the scheme in terms of reduced carbon emissions. Data were collected weekly and grouped by months; a week was considered to be part of the month if the week ended within that month.

To calculate the theoretical amount of remaining propellant in recycled pMDIs (based on the expected overfill included by the manufacturer), the following assumptions were made for the time of return: each pMDI will have emitted all of the number of doses as per the label claim; the pMDI will not have been used beyond the claimed number of doses; and storage of the pMDI will have been as per the manufacturer’s instructions.

Modelling considered the following factors for a number of commercialised pMDIs that may be returned in the scheme: the type of propellant (HFA 134a or HFA 227ea); the weight proportion of the contents made up by the propellant if cosolvents were used; the label claim number of doses; the impact of time from manufacture to account for propellant loss due to leakage; and considerations for initial manufacturing overfill. Considerations for overfill included the following: allowances for leakage over the total shelf life of the pMDI; filling process tolerances; metering valve tolerances; non-accessible volume (the amount required to reach the filling point of the metering valve); required propellant conversion from liquid to vapour used to maintain equilibrium inside the pMDI; manufacturing process requirements (such as test actuations); and dose counter accuracy (developed to avoid undercount).

As far as possible, values were verified by reviewing information in the public domain, including approved labels and regulatory reviews. This approach provided an indicative value for the potential residual propellant in a pMDI that had been manufactured as intended, used to its full life as intended, and promptly returned to the waste management company. The model could not account for pMDI propellant residual for the following: pMDIs returned unused or partly used; pMDIs significantly past their expiry date; or pMDIs operated beyond the label claim number of actuations.

### Carbon dioxide equivalencies

Carbon dioxide savings were estimated from F-gas quantities using the UK government online conversion tool^19^. Carbon dioxide saving equivalencies in the context of sequestration by trees were estimated using the United States Environmental Protection Agency online tool^50^. This tool was chosen because it is simple to use and facilitates conversion from standardised emissions values to everyday scenarios with which the public are familiar.

### Patient questionnaire

Patients could scan the QR code in the information leaflet included in the envelope to access the feedback questionnaire. This was a qualitative survey for patients involved in the Take AIR scheme to provide their anonymised opinion in a multiple-choice online feedback form. There was no active recruitment for the questionnaire; any patient with an envelope could choose to participate.

### Reporting summary

Further information on research design is available in the Nature Research Reporting Summary linked to this article.

## Supplementary information

Reporting Summary

## Data Availability

The data sets generated and/or analysed during the current study are available from the corresponding author on reasonable request.
