# Supplementary material for: Understanding the feasibility and environmental effectiveness of a pilot postal inhaler recovery and recycling scheme
Source: NPJ Prim Care Respir Med. 2023 Jan 21;33:5. doi: 10.1038/s41533-023-00327-w (PMC9864496; doi:10.1038/s41533-023-00327-w)
Supplement: Supplementary file 1 — Reporting Summary [file 41533_2023_327_MOESM1_ESM.pdf]

nature portfolio

Corresponding author(s): Anna Murphy

Last updated by author(s): Nov 4, 2022

Reporting Summary

Nature Portfolio wishes to improve the reproducibility of the work that we publish. This form provides structure for consistency and transparency in reporting. For further information on Nature Portfolio policies, see our Editorial Policies and the Editorial Policy Checklist.

Statistics

For all statistical analyses, confirm that the following items are present in the figure legend, table legend, main text, or Methods section.

n/a	Confirmed	
☒	☐	The exact sample size (*n*) for each experimental group/condition, given as a discrete number and unit of measurement
☒	☐	A statement on whether measurements were taken from distinct samples or whether the same sample was measured repeatedly
☒	☐	The statistical test(s) used AND whether they are one- or two-sided<br>*Only common tests should be described solely by name; describe more complex techniques in the Methods section.*
☒	☐	A description of all covariates tested
☒	☐	A description of any assumptions or corrections, such as tests of normality and adjustment for multiple comparisons
☒	☐	A full description of the statistical parameters including central tendency (e.g. means) or other basic estimates (e.g. regression coefficient) AND variation (e.g. standard deviation) or associated estimates of uncertainty (e.g. confidence intervals)
☒	☐	For null hypothesis testing, the test statistic (e.g. *F*, *t*, *r*) with confidence intervals, effect sizes, degrees of freedom and *P* value noted<br>*Give P values as exact values whenever suitable.*
☒	☐	For Bayesian analysis, information on the choice of priors and Markov chain Monte Carlo settings
☒	☐	For hierarchical and complex designs, identification of the appropriate level for tests and full reporting of outcomes
☒	☐	Estimates of effect sizes (e.g. Cohen's *d*, Pearson's *r*), indicating how they were calculated

Our web collection on statistics for biologists contains articles on many of the points above.

Software and code

Policy information about availability of computer code

Data collection	Microsoft Excel 365
Data analysis	Microsoft Excel 365

For manuscripts utilizing custom algorithms or software that are central to the research but not yet described in published literature, software must be made available to editors and reviewers. We strongly encourage code deposition in a community repository (e.g. GitHub). See the Nature Portfolio guidelines for submitting code & software for further information.

Data

Policy information about availability of data

All manuscripts must include a data availability statement. This statement should provide the following information, where applicable:

- Accession codes, unique identifiers, or web links for publicly available datasets
- A description of any restrictions on data availability
- For clinical datasets or third party data, please ensure that the statement adheres to our policy

The data sets generated and/or analysed during the current study are available from the corresponding author on reasonable request.

Human research participants

Policy information about studies involving human research participants and Sex and Gender in Research.

Reporting on sex and gender	Not relevant – manuscript reports inhaler recycling scheme, not a human research study
Population characteristics	Not relevant – manuscript reports inhaler recycling scheme, not a human research study
Recruitment	Not relevant – manuscript reports inhaler recycling scheme, not a human research study
Ethics oversight	Not relevant – manuscript reports inhaler recycling scheme, not a human research study

Note that full information on the approval of the study protocol must also be provided in the manuscript.

Field-specific reporting

Please select the one below that is the best fit for your research. If you are not sure, read the appropriate sections before making your selection.

☐ Life sciences ☐ Behavioural & social sciences ☒ Ecological, evolutionary & environmental sciences

For a reference copy of the document with all sections, see nature.com/documents/nr-reporting-summary-flat.pdf

Ecological, evolutionary & environmental sciences study design

All studies must disclose on these points even when the disclosure is negative.

Study description	The study describes results from the Take AIR scheme, designed and funded by Chiesi, with a primary aim of developing and assessing the feasibility of a postal option for patients to return inhalers for recycling, as an alternative to the community pharmacy waste collection service.
Research sample	All community pharmacies in the Leicester, Leicestershire and Rutland (LLR) region (a total of 227) and three hospitals at the University Hospitals of Leicester (UHL) NHS Trust were invited to participate in the Take AIR scheme. This was for the purposes of a regional pilot study to explore feasibility. Envelopes were offered to any patients interested in returning inhalers via the scheme, with no restrictions.
Sampling strategy	No sample size calculations were performed. The scheme was designed to explore a postal inhaler recycling scheme and evaluate uptake by patients over a 12-month period (from 5 February 2021 to 18 February 2022). Data on numbers and types of inhalers returned were collected.
Data collection	The waste management partner (Grundon) collected data on inhalers being returned and recorded weekly totals.
Timing and spatial scale	The results provided here describe findings from a 12-month period (February 2021-February 2022). Data on returned inhalers were collected weekly and grouped by months for analysis.
Data exclusions	No data were excluded from the analyses.
Reproducibility	Not applicable for this study type - not an experimental study
Randomization	Not applicable for this study type - not an experimental study
Blinding	Not applicable for this study type - not an experimental study

Did the study involve field work? ☐ Yes ☒ No

Reporting for specific materials, systems and methods

We require information from authors about some types of materials, experimental systems and methods used in many studies. Here, indicate whether each material, system or method listed is relevant to your study. If you are not sure if a list item applies to your research, read the appropriate section before selecting a response.

Materials & experimental systems

n/a	Involved in the study	
☒	☐	Antibodies
☒	☐	Eukaryotic cell lines
☒	☐	Palaeontology and archaeology
☒	☐	Animals and other organisms
☒	☐	Clinical data
☒	☐	Dual use research of concern

Methods

n/a	Involved in the study	
☒	☐	ChIP-seq
☒	☐	Flow cytometry
☒	☐	MRI-based neuroimaging
